# Food Security Dimensions in US Disaster Plans: A Comparative Analysis of States and Territories

**DOI:** 10.1017/dmp.2025.98

**Published:** 2025-04-24

**Authors:** Maria M. Muñoz, Danielle J. Gartner, Saria Hassan, Melissa Fuster

**Affiliations:** 1 Department of Social and Behavioral Population Sciences, Tulane University Celia Scott Weatherhead School of Public Health and Tropical Medicine, New Orleans, LA, USA; 2 Department of Medicine, Emory University School of Medicine, Atlanta, GA, USA

**Keywords:** climate-induced disasters, food security, disaster food security framework, disaster plans, United States

## Abstract

**Objective:**

Disaster response plans play a major role in mitigating the impact of climate-related disasters on community food access. This study examined existing disaster response plans in 5 US locations that experienced the costliest hurricanes since 2017 (states: Florida, Texas, Louisiana; territories: Puerto Rico, US Virgin Islands) to assess how existing disaster response plans and response efforts address food-related issues across 4 key domains: availability, accessibility, agency, and acceptability.

**Methods:**

A content analysis of disaster response plans was conducted. Disaster response plans were complemented by a review of gray literature and media sources examining the post-hurricane aftermaths. Disaster plans were coded using a deductive analysis approach guided by the Disaster Food Security Framework.

**Results:**

The analysis revealed significant disparities in planning and resources between territories and states. Findings highlight political and structural drivers of disparities in food access, particularly in US territories. State-mandated procedures resulted in a consistent level of effectiveness in their food distribution strategies.

**Conclusions:**

These disparities underscore the need for targeted policy reforms and enhanced federal support to ensure equitable food security during disasters.

Disasters occur when a community or population lacks the capacity to manage the hazard’s effects with the available resources at their disposal.^1,2^ Climate-related disasters are escalating rapidly worldwide, increasing in frequency, severity, and geographic distribution.^3,4^ The United States (US) has incurred 190 separate billion-dollars in disaster-related damages from 2015 to 2024, resulting in over 6300 fatalities and an estimated $1.4 trillion in total economic losses.^4^ Among these events, tropical cyclones (including hurricanes) have caused the greatest financial impact, with cumulative losses totaling $1543.2 billion and an average cost of $23.0 billion per event.^4^ These storms severely disrupt critical infrastructure, particularly food systems, by impacting food availability, accessibility, and distribution, further exacerbating vulnerabilities in affected communities.^5,6^ Beyond immediate disruptions, disasters have lasting social, environmental, and economic repercussions,^2^ underscoring the urgent need for effective disaster response and resilience strategies.^4,7–16^

The Food and Agriculture Organization (FAO) defines food security as the “condition in which all people, at all times, have both physical and economic access to sufficient, safe, and nutritious food that aligns with their dietary needs and preferences, promoting an active and healthy life.”^17^ To be food secure, 4 dimensions—availability, accessibility, utilization, and stability—must be met simultaneously.^17^ Disasters elevate the risk of food insecurity, particularly for households experiencing financial strain, housing instability, or shifts in composition.^18^ Given the widespread impacts,^19,20^ effective policies and regulations are crucial to ensuring post-disaster access to adequate nutrition. Without such measures, prolonged food supply disruptions could exacerbate health risks, deepen disparities, and destabilize local food systems.^21^

In the US and its territories, the 1990 National Nutrition Monitoring and Related Research Act mandates food assistance align with federal Dietary Guidelines for Americans.^13^ However, prior to Hurricane Katrina (2005), many states lacked adequate disaster response plans. Katrina’s aftermath prompted the Post-Katrina Emergency Management Reform Act of 2006, restructuring the Federal Emergency Management Agency (FEMA) and leading to the creation of the National Response Framework (NRF),^22^ a comprehensive federal disaster response guide.^23,24^ The NRF coordinates across local, state, and federal authorities, non-governmental organizations (NGOs), faith-based groups, and private businesses. Under this framework, the United States Department of Agriculture (USDA) leads the Emergency Support Function-11, overseeing disaster nutrition assistance. Another critical reform, the Stafford Act, grants the Secretary of Agriculture the authority to implement the Disaster Supplemental Nutrition Assistance Program (D-SNAP) with a presidential major disaster declaration. If enacted, D-SNAP provides eligible survivors 1 month of financial benefits that can be used to purchase food, except in extraordinary circumstances. State agencies evaluate the need for D-SNAP or other feeding programs after disasters and submit detailed requests to Food and Nutrition Service (FNS), which offers disaster nutrition assistance through 3 methods.^23,24^ (Figure 1).

Although the NRF and Stafford Act provide general guidance, each state develops its own disaster response plan, which may result in wide variations in food security after hurricanes. Yet, despite the critical role of food and nutrition assistance in disaster response,^25^ there is scant evidence on how food distribution functions in the aftermath of disasters across states and territories. Understanding these differences is essential for developing disaster response plans that effectively mitigate food insecurity amid disasters, particularly among vulnerable populations. However, the US lacks a strong evidence base on how to effectively meet food and nutrition needs in disaster response. Without data-driven strategies, policies risk overlooking vulnerable populations or distributing food that fails to meet actual needs.

This study sought to examine how locality-specific disaster response plans address food security in hurricane-prone US states and territories through the following research objectives: (1) analyze variations in food distribution strategies between Florida, Texas, Louisiana, Puerto Rico (PR), and the US Virgin Islands (USVI) disaster response plans; (2) identify disparities in disaster food response plans; and (3) highlight areas for policy and programmatic improvement. To achieve these objectives, this study is guided by the following research question: How do disaster response plans and media coverage address the 4 dimensions of food security following hurricanes, and how do food distribution strategies compare between US states and territories? The South, Central, and Southeast US, including the Caribbean territories, have suffered the highest cumulative disaster costs, reflecting their exposure to extreme weather events.^4^ Florida leads the US in total disaster costs ($450 billion), followed by Texas ($436 billion), and Louisiana (~$314 billion).^4^ PR and the USVI have also faced severe economic and infrastructural losses, particularly following the devastating 2017 hurricane season, which was the costliest on record, exceeding $300 billion in damages.^4,26^ Findings from this study will contribute to the development of evidence-based recommendations to enhance disaster preparedness and response efforts, ensuring equitable access to nutritious food in disaster-affected communities.

## Methods

A comparative content analysis was conducted, utilizing a deductive coding approach guided by the Disaster Food Security Framework (DFSF), a theoretical model developed for assessing food insecurity in disaster settings.^27^ The DFSF comprises 4 key domains—availability, accessibility, agency, and acceptability—that collectively influence food security in disaster contexts. This framework was chosen because it is the only model specifically designed to assess food security in the context of disasters, making it uniquely suited for this study. Unlike broader food security models, which often focus on chronic food insecurity, DFSF directly accounts for disruptions in food access, distribution networks, and emergency food assistance—critical elements in disaster response. *Availability* categorizes food accessible through mainstream sources, including grocery stores and food provided through assistance and donation programs. This is crucial, given that food assistance programs, food banks, food pantries, and other donation approaches constitute vital sources of food in post-disaster settings. *Accessibility* addresses economic, physical, and social access, emphasizing affordability, the use of the D-SNAP, transportation, distribution networks, number of food sources, and safety practices. *Agency* focuses on households’ ability to prepare and safely store food, considering factors such as equipment (e.g., stoves, sinks, refrigerators, and freezers), services (e.g., electricity, water, and waste disposal), and other necessities (e.g., ingredients and a functional kitchen). *Acceptability* addresses individual health needs, dietary restrictions, preferences, and nutrition aspects, including food quantity, quality, and safety.^27^ The DFSF was utilized to assess state-mandated policies and procedures. The focus was on Florida, Texas, and Louisiana—and US Territories, PR, and the USVI—as these localities have experienced the costliest hurricanes since 2017.^28^

The main unit of analysis was each locality’s disaster response plan, sourced from official state and territorial websites, where available. These documents outline detailed response strategies, agency responsibilities, and implementation procedures. Comprehensive state-level disaster plans were accessible for Florida, Texas, and Louisiana, whereas equivalent documents were limited or unavailable for PR and USVI. Given the variability in available plans, the analysis was supplemented with gray literature and media sources (Table 1) to capture disaster food security efforts in contexts where official plans were unavailable, particularly in PR and USVI. Media sources were selected to provide real-time insights into post-disaster food security challenges, particularly where government-issued documents lacked details. To mitigate potential biases, sources were drawn from a diverse range of organizations, including FEMA, USDA, NGOs (relief organizations, food banks, and service providers), and major news outlets. A systematic online search was conducted using Google, applying predefined search term combinations by location (“hurricane,” “food distribution,” “food insecurity”) and restricting results to the first 3 pages per search. The search focused on major hurricanes affecting the region since 2017, namely Hurricanes Ian (2022), Harvey (2017), Ida (2021), and Maria (2017). Exclusions were made for sources lacking relevant content, unavailable weblinks, and repetitive information. In cases where a source contained insights relevant to multiple locations, it was referenced across different study sites.

The study methodology follows guidelines from the Consolidated Criteria for Reporting Qualitative Research (COREQ), ensuring rigor, transparency, and consistency in qualitative coding and reporting.^29^ Data sources were coded using NVIVO and MAXQDA to ensure a structured and systematic qualitative analysis. The DFSF framework guided the initial coding process, organizing data into predefined categories related to the 4 dimensions. To ensure methodological rigor and inter-coder reliability, the analysis was conducted by 2 independent coders, with coding discrepancies resolved by consensus through regular calibration meetings. In cases where consensus was not immediately achieved, a third senior reviewer adjudicated differences to ensure consistency in coding application. Additionally, emerging themes were discussed with the broader research team to validate interpretations. As this research involved the review of public documents, IRB approval was not required. This integrated methodological approach, combining official disaster response plans, gray literature, and media analysis, provides a comprehensive understanding of food security planning, illuminating nuances within the broader disaster response context.

## Results

Table 2 presents selected characteristics of the localities included in this study, showcasing key contextual differences in food security and disaster response. The US territories show the greatest prevalence of poverty and food insecurity,^30^ with the USVI experiencing a food insecurity rate more than double that of Louisiana, the most food insecure US state in this study. Despite the USVI and PR both being US territories, differences in policy implementation, notably, food assistance and maritime laws (e.g., Jones Act), influence disaster food security responses differently. These factors are further addressed in the next section.

The analysis revealed that food availability, accessibility, and acceptability during disaster periods were the primary themes emphasized in disaster plans and media communications. Few disaster plans addressed agency (Table 3, see Supplemental File for more details). While US states prioritized coordinated food distribution strategies and infrastructure resilience, PR and the USVI faced significant deficiencies across all DFSF domains, particularly in accessibility and agency. The following examines each dimension separately, concluding with an analysis of how these dimensions interact.

### Availability

State response plans comprehensively addressed all subdomains of food availability, ensuring adequate supply at distribution points such as grocery stores and emergency food aid programs. In contrast, US Territories lacked adequate support for the supply and provision of food. Donation assistance was emphasized in state response plans and recognized as crucial in US territories by media accounts, with local and national food banks playing a central role in storing and supplying food aid in regions with high rates of food insecurity, such as Louisiana, PR, and the USVI (Table 3). There were notable differences between states and territories for supply and provision at food distribution points. State response plans emphasized coordination and cross-state collaboration. Texas and Louisiana, for example, established dedicated food teams at the local level for emergency food response during disasters. Texas coordinated with national food networks to emphasize stockpiling for sustained provision, while Louisiana’s plan detailed inventory management and a 3-phase timeline for food provision, including partnerships with food service providers. In contrast, PR and the USVI lacked food stockpiling measures, making them heavily reliant on FEMA distribution sites post-disaster. Unlike US states that could restock supplies via road networks, PR and the USVI required shipments by air and sea. Media documents highlighted that shipments were often delayed as a result of logistical inefficiencies and maritime restrictions. The Jones Act, which requires that goods transported between US ports be carried on US-built and US-operated vessels, exacerbated supply delays despite waivers granted post-disasters.^37–39^ Even with waivers, inefficiencies persisted, with average delivery times reaching 69 days for PR,^40,41^ leaving only a 30-day grocery supply.^42^ Although the USVI is exempt from the Jones Act, limited port capacity and resource competition between relief and commercial supply chains created additional delays.

### Accessibility

Disaster response plans prioritized economic and physical accessibility by integrating financial food aid programs, like D-SNAP and FNS waivers, to alleviate income changes and support affected households in the aftermath of disasters.^25^ Florida implemented a hybrid D-SNAP model, combining in-person and virtual enrollment, and the USDA issued SNAP benefits in advance of Ian’s landfall to provide early financial relief. Similarly, Texas’ plan facilitated access to food assistance programs through information referral networks. A key contrast in this area was the case of PR. Unlike SNAP, which provides automatic funding increases based on need, PR relies on the Nutrition Assistance Program (NAP), a block grant with a fixed maximum allocation. As a result, benefit amounts are limited based on a projection of the number of participants, which may not accurately reflect need in times of disasters. NAP has no equivalent to D-SNAP; therefore, disasters or emergencies require new appropriations by Congress. After Hurricane Maria, PR residents waited 6 months for additional food aid, highlighting a major accessibility gap.^43^ In the USVI, media reports underscored challenges in D-SNAP implementation, compounded by power and communication outages that prevented electronic food purchases. Physical accessibility was addressed through mobile feeding units and expanded food distribution sites in state plans. Louisiana prioritized cross-agency coordination to deploy supplies, while PR and the USVI struggled with port congestion, leaving food undistributed despite availability. Social factors such as trust, worry, fear, stigma, or coping behaviors were not directly accounted for in the disaster plans.

### Agency

Agency, the ability of individuals to prepare, store, and utilize food, was almost entirely absent from state and territory response plans. Only Texas explicitly addressed agency. Texas focused on infrastructure only, and emphasized the restoration of utilities to enable residents to cook meals at home and purchase food in stores. In contrast, Florida, Louisiana, and US territories relied on NGOs and local restaurant coalitions to provide ready-to-eat meals, as opposed to the provision of infrastructure or measures to augment self-efficacy. The lack of attention to agency has significant long-term implications, particularly in fostering dependency on external aid rather than promoting resilience and autonomy. In PR, for example, post-Hurricane Maria recovery efforts prioritized short-term food distribution without parallel investments in local food systems or household-level food preparation infrastructure. Media reports from this period indicated limited community capacity to transition from emergency relief to self-sufficiency. Similarly, in the USVI, extended reliance on mass feeding sites highlighted the absence of disaster plans supporting residents’ ability to store and prepare their own food, exacerbating vulnerabilities when external aid diminished.

### Acceptability

All state disaster plans addressed nutrition. They primarily focused on food and water safety. Additional considerations included providing nutritionally appropriate food and addressing the specific nutritional needs of various populations (infants, children, and those receiving meals from mass feeding sites). While plans briefly addressed needs (i.e., individual dietary requirements), no locality explicitly mentioned medical, health, religious, or cultural preferences. Florida’s disaster plan prioritized providing food for mass feeding sites using the USDA foods inventory. In contrast, Texas briefly mentioned dietary considerations and collaboration on feeding menus. Media coverage of Texas emphasized efforts to provide nutritious foods. Louisiana’s plan referenced nutritional guidelines and aimed to meet specific dietary requirements. In PR, the nutritional quality of food aid was questioned after Hurricane Maria due to the presence of highly processed foods with low nutritional value. Issues concerning food preferences and specific dietary needs were not acknowledged.^13^ Similarly, in the USVI, nutritional aspects and specific preferences were not comprehensively addressed.

### Interconnectedness of the 4 Dimensions

The 4 dimensions of food security (availability, accessibility, agency, and acceptability) are not isolated; they are deeply interconnected, particularly in disaster contexts where disruptions in one domain often trigger cascading effects in others. For instance, when food availability is limited, as observed in PR and the USVI, this scarcity directly exacerbates accessibility challenges. Disruptions in supply chains, damaged transportation infrastructure, and economic shocks can create both physical and financial barriers that prevent communities from obtaining essential food supplies. These accessibility constraints further undermine agency. Without reliable access, households lose the capacity to make informed choices about their food, especially when critical infrastructures such as electricity, refrigeration, and safe water are compromised. This lack of agency means that even if food is eventually available, households may not be able to store or prepare. The inability to store and prepare food further exacerbates food insecurity, making it more difficult to meet dietary and nutritional needs. Furthermore, when agency is diminished, individuals are often forced to consume whatever food is available, regardless of whether it aligns with their cultural, dietary, or nutritional preferences. Thus, further compromising acceptability. This forced compromise not only impacts immediate well-being but can also have long-term effects on health and community resilience.

## Limitations

Limitations of the study include incomplete information for Texas due to ongoing revisions of the state’s disaster plans. The Texas disaster response plan used for these analyses may not be up to date with the revised plan. However, results still yield informative information on the state of disaster response plans overall, particularly as it relates to differences between US states and territories. To our knowledge, PR and the USVI lack locality-level disaster response plans. Media reports were used to supplement the unavailable response plans for PR and the USVI. To this end, the study’s source procurement through the online search engine relies on its algorithm, which can vary and affect the search results. Another consideration is the application of the DFSF, as one originating from an individual perspective, being applied to plans established at the community level, introducing a potential gap between individual-level considerations and community-level planning dynamics. Nevertheless, the framework was used because it provides a comprehensive method for assessing disaster impact on food security, which is essential for creating comparable and actionable insights across different regions.

## Discussion

The application of the DFSF framework provided a structured approach for examining disaster food-related planning, revealing substantial differences between US states and territories. While most states focused on availability, accessibility, and acceptability, they largely neglected agency, limiting long-term recovery efforts. In contrast, PR and the USVI faced systemic barriers, including insufficient federal support, infrastructure limitations, and reliance on external aid. This led to delays in food assistance and greater dependence on external relief efforts. Despite common reliance on local food banks, variations in coordination, partnerships, and logistical capacity reflected the nuanced landscape of disaster and response across these locations.

These findings were generally consistent with existing research on food security in disaster settings, which highlights the prioritization of short-term food availability over long-term resilience.^8,9,14,22,42^ Prior studies on Hurricane Katrina similarly found that emergency food responses emphasized immediate distribution rather than strategies for rebuilding food security through self-sufficiency strategies.^15,44^ While DFSF was initially tested in the context of the COVID-19 pandemic, this study extends its application to hurricanes, reinforcing its adaptability across different disaster contexts. Insights gained from this application can guide further exploration of the model across other disaster types. However, the DFSF lacks a temporal dimension, limiting its ability to address the evolving needs of communities during prolonged recovery periods. This study found that while availability and accessibility were prioritized in early response efforts, agency and self-sufficiency were overlooked in long-term recovery planning. Future adaptations of DFSF should integrate phased recovery planning, ensuring that food security strategies evolve from immediate relief to sustainable food access.

While state disaster plans accounted for food availability and accessibility within FEMA’s phased recovery framework, they remained largely reactive for PR and the USVI. Thus, plans failed to address infrastructure and supply chain fragility that continue to increase residents vulnerability to food access disparities during disasters.^45^ As climate change drives more frequent and severe disasters, food security programs and policies must extend beyond short-term aid and integrate resilience-building strategies. Unlike states with more robust infrastructure and supply networks, PR and the USVI face persistent food insecurity due to import dependency, logistical barriers, and the absence of localized food systems. Strengthening localized food systems and enhancing emergency stockpiling capacity are critical steps for reducing disaster-related food insecurity in these regions. The relationship between climate change and food security must be addressed in disaster planning. Rising temperatures, extreme weather events, and shifting precipitation patterns continue to disrupt agricultural production, supply chains, and post-disaster recovery.^10^ PR and the USVI are especially vulnerable due to aging infrastructure, geographic isolation, and limited emergency preparedness resources.^45^ The increasing unpredictability of climate-related disasters calls for a shift from relief-based approaches to preemptive food security planning that prioritizes regional self-sufficiency and adaptive capacity. While US policies focus primarily on relief, international frameworks such as the FAO’s Disaster Risk Reduction for Food Security and the Sphere Standards prioritize climate resilience, localized food production, and cross-sector coordination.^46,47^ Incorporating best practices from these models could improve disaster food security planning in the US, particularly by integrating climate adaptation strategies into federal emergency management frameworks.

Policy reforms and targeted interventions are necessary to address structural inequities in disaster response plans and food distribution, particularly PR’s reliance on the block-grant-based NAP. At the time of writing, 2 bills, H.R. 253 and S. 949, are under consideration in Congress. If passed, these bills would transition PR from the NAP to SNAP, addressing systemic issues with NAP block grant funding. The bill is being supported by a coalition of over 80 organizations, including private industry, national nonprofits, academic think tanks, and religious groups, advocating for these provisions to be included in the upcoming Farm Bill.^48–50^ Beyond the NAP-SNAP transition, additional policy reforms should mandate investment in disaster-resilient food infrastructure, such as expanding cold storage facilities and reinforcing local procurement systems in hurricane-prone areas.

Disaster food security planning in US territories must align with comprehensive and structured planning approaches used in US states. Ensuring equitable access to food resources during crises requires emergency response plans that prioritize localized food networks, regional supply chain resilience,^7,12^ and flexible aid programs tailored to the severity of disasters.^51^ Improved coordination between federal, state, and non-governmental entities is essential for enhancing food distribution efficiency and post-disaster recovery. Strengthening localized food networks, expanding emergency stockpiling, and enhancing intergovernmental collaboration will be crucial in building resilient and equitable disaster food security system.

## Conclusions

Ensuring equitable disaster food security requires stronger planning, improved coordination, and targeted policy reforms. While disaster food response efforts address immediate food availability and accessibility, long-term recovery strategies must prioritize self-sufficiency, infrastructure resilience, and localized food networks. Policy-makers must integrate all 4 dimensions of the DFSF, particularly agency, which remains the most overlooked in disaster planning. Structural inequities in food assistance programs, particularly PR’s reliance on NAP rather than SNAP, highlight the need for policy reforms that eliminate bureaucratic delays and improve food aid accessibility post-disaster. Expanding localized food production and emergency stockpiling in disaster-prone regions can further enhance resilience. As climate-related disasters continue to intensify, proactive, resilience-based planning is essential for minimizing long-term vulnerabilities and ensuring food security for affected populations. Further research is needed to explore how different policy frameworks within the US influence disaster response effectiveness, particularly regarding food distribution, resource allocation, and equity-driven reforms. Longitudinal studies are essential to understand the duration and intensity of food insecurity challenges faced by affected populations, helping determine the appropriate scope and immediacy of required food assistance. Strengthening disaster food security policies through equity-driven reforms, improved federal coordination, and climate-conscious planning will be critical to ensure all communities, especially the most vulnerable, are better prepared for future crises.

## Supplementary Material

supplementary material

**Supplementary material.** The supplementary material for this article can be found at http://doi.org/10.1017/dmp.2025.98.

## Figures and Tables

**Figure 1.**
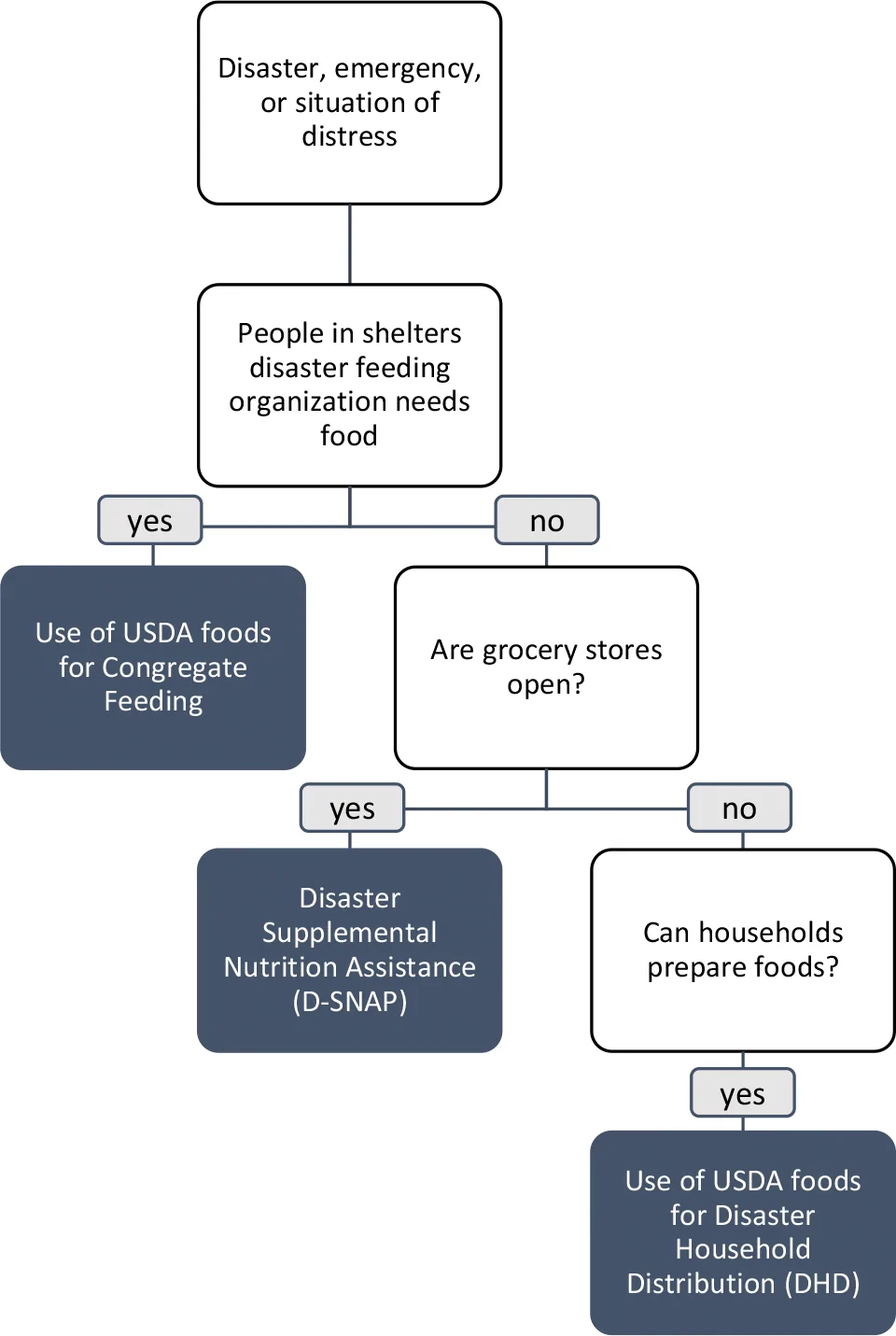
Food and Nutrition Service (FNS) disaster services: overview of assistance provided to affected populations. *Source:* Authors’ modified version of the original exhibit in Food and Nutrition Service: USDA Foods in Disaster Manual.^23^

**Table 1.** Selected characteristics of media sources in the specified states and territories (2017–2022)

	Florida	Texas	Louisiana	Puerto Rico	US Virgin Islands
Source type & qty (n = 127)					
FEMA Website	0	1	0	3	2
News	26	17	12	17	10
NGO (relief organizations)	4	2	0	4	2
NGO (food banks)	6	4	4	5	0
NGO (service)	1	1	0	1	2
USDA website	2	3	2	1	2
Search term combinations	“Florida AND Hurricane Ian AND Food Distribution”, “Florida AND Hurricane Ian AND Food Insecurity”	“Texas AND Hurricane Harvey AND Food Distribution”, “Texas AND Hurricane Harvey AND Food Insecurity”	“Louisiana AND Hurricane Ida AND Food Distribution”, “Louisiana AND Hurricane Ida AND Food Insecurity”	“Puerto Rico AND Hurricane Maria AND Food Distribution”, “Puerto Rico AND Hurricane Maria AND Food Insecurity”	“US Virgin Islands AND Hurricane Maria AND Food Distribution”, “US Virgin Islands AND Hurricane Maria AND Food Insecurity”

*Source:* Authors’ analysis of data from media sources, 2017–2022.

**Table 2.** Case descriptions of specified states and territories

Status	Florida	Texas	Louisiana	Puerto Rico	US Virgin Islands
	State	State	State	US Territory	US Territory
persons in poverty (percent)	12.7	14.0	18.6	41.7∥	22.9
Prevalence of household-level food insecurity (percent)	11.4	15.5	15.2	31.2Ɨ	39.2Ɨ
USDA food assistance	SNAP	SNAP	SNAP	NAP	SNAP
Jones Act status	Applies	Applies	Applies	Applies	Does not apply
Hurricane and year	Hurricane Ian (2022)	Hurricane Harvey (2017)	Hurricane Ida (2021)	Hurricane Maria (2017)	Hurricane Maria (2017)
Cost of damages	$112.9 billion	$151.3 billion	$75 billion	$108.9 billion (collectively between PR and USVI)	
Main agency	Florida’s State Emergency Response Team (SERT)	Texas Division of Emergency Management (TDEM)	Governor’s Office of Homeland Security and Emergency Preparedness	Puerto Rico Emergency Management, known as Negociado para el Manejo de Emergencias y Administracion de Desastres (NMEAD)	Virgin Islands Territorial Emergency Management Agency (VITEMA)
Official documents *supplement	State of Florida 2020 Comprehensive Emergency Management Plan	State of Texas Emergency Management Plan 2020	State of Louisiana Emergency Operations Plan 2019 State of Louisiana Mass Care Feeding Plan*		
Date released	2020	2020	2022; 2019*		
Length (pages)	240 pp.	51 pp.	157 pp.; 20 pp.*		
Emergency Support Function (ESF-#) reviewed	ESF–6 (Mass Care) ESF–11 (Food & Water) ESF–15 (Volunteer & Donations)	ESF–6 (Mass Care)	ESF–6 (Mass Care, Housing, and Human Services Annex) ESF–11 (Agricultural Annex)		

*Source:* Persons in poverty (percent) retrieved from US Census Bureau, American Community Survey 2022.^31^

∥ Persons in poverty (percent) retrieved from US Census Bureau, Puerto Rico Community Survey 2022.^32^ Prevalence of household-level food insecurity, average 2020–2022 (percent) data comes from USDA ERS – Key Statistics & Graphics.^33^ Totals exclude households for which food security status is unknown because household respondents did not give a valid response to any of the questions in the food security scale. These exclusions represented about 0.2 percent of all households in 2020, 0.2% in 2021, and 0.2% in 2022.

Ɨ Prevalence of household-level food insecurity, average 2020–2022 (percent) data comes from a cross-sectional analysis of baseline ECHORN cohort study data between 2013 and 2018 (*n* = 1939).^30^ This paper emphasizes SNAP and NAP due to the provision of temporary food assistance benefits to eligible households affected by natural disasters through DSNAP.^34,35^ Jones Act Status retrieved from the US Customs and Border Protection.^36^ Hurricanes mentioned are among the most recent and devastating.^28^

**Table 3.** Analysis of existing disaster response plans and post-disaster response efforts addressed food security in affected areas (2017–2022)

		Availability		Accessibility			Agency		Acceptability	
		Supply/Provision Failure	Donation Assistance	Economic	Physical	Social	Infrastructure	Self-Efficacy	Nutrition	Needs/Preferences
		Grocery stores, supermarkets, farms, gardens, restaurants	Food bank, food pantry, school meal, other in-kind food programs	Affordability, income changes, financial food aid (e.g. SNAP)	Transportation, distribution networks, number of food sources, safety practices	Experiences e.g., trust, worry, fear, uncertainty, stigma, coping behaviors	Kitchen facilities, equipment, space, services, ingredients	Food preparation/cooking ability, knowledge to use foods, desire, confidence in meal preparation	Nutritional value e.g., processed foods, low fruit/vege intake, variety/diversity, quality, and safety of foods available and accessible to households	Medical, health, religious, cultural, ethical, local, organic, food that can be stored and prepared
States	Florida	✓	✓	✓	✓				✓	✓
	Texas	✓	✓	✓	✓		✓		✓	✓
	Louisiana	✓	✓	✓	✓				✓	✓
U.S. Territories	Puerto Rico		✓							
	U.S. Virgin Islands		✓	✓						

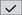
Food Security dimension is addressed in both state-mandated disaster plans and media coverage.

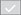
Food Security dimension is addressed only in media coverage.

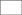
Food Security dimension is not mentioned or covered in either disaster plans or media coverage.

Analysis of Existing Disaster Response Plans and Post-Disaster Response Efforts Addressed Food Security in Affected Areas (2017-2022)

Source/Notes: Authors’ analysis of data from state disaster response plans and state and territory media sources, 2017–2022
