# Supplementary material for: Food Security Dimensions in US Disaster Plans: A Comparative Analysis of States and Territories
Source: Disaster Med Public Health Prep. Author manuscript; Available in PMC 2026 Oct 5. (PMC13635830; doi:10.1017/dmp.2025.98)
Supplement: Supplementary file 1 [file NIHMS2205156-supplement-supplementary_material.docx]

**Food Security Dimensions in U.S. Hurricane Disaster Plans: A Comparative Analysis of States and Territories**

Maria M Munoz, Danielle J Gartner, Saria Hassan, Melissa Fuster

**DETAILED FOOD SECURITY DIMENSIONS IN DISASTER PLANS**

| **States / U.S. Territories** | **Availability** | **Accessibility** | **Agency** | | **Acceptability** |
| --- | --- | --- | --- | --- | --- |
| **Florida** | The disaster plan integrated donation assistance by outlining coordination roles among local food banks, food pantries, school meals, and other in-kind food programs within the Feeding Florida network. These roles involved emergency mass feeding preparation, distribution, and soliciting donated groceries.  Regarding supply and provision, the plan also incorporated public and private partnerships between food banks, churches, and service providers to supplement the USDA food inventory. It included details on stockpiling food and coordinating with neighboring states. | The disaster plan integrated financial food aid (e.g. DSNAP, WIC, FNS waivers) to tackle affordability concerns, income changes, and child nutrition programs resulting from disasters.  The plan addressed the physical accessibility of transportation, distribution networks, and the number of food sources. Coordination with public and private agencies (e.g., national NGOs, commercial food vendors) was outlined to determine the required amount of food and provide feeding and hydration along evacuation routes. It identified and assessed food requirements on a two-phase basis, addressing critical emergency needs and long-term sustained needs.  Social accessibility regarding trust, worry, fear, stigma, or coping behaviors were not directly addressed. | Instead of focusing on restoring individuals' infrastructure or self-efficacy in food preparation, disaster plans integrated mobile kitchens and equipment. These plans included kitchen facilities, coordination for non-USDA foods, and deployment of "Strike Teams" with skilled personnel and mobile kitchens with full-time cooks for quality and food safety. However, specifics on space and ingredients were not addressed.  Self-efficacy in food preparation and cooking ability, their knowledge of food usage, or their desire and confidence in meal preparation were not directly addressed. | | The disaster plan referenced a "Meal Compilation Tool" to address the nutritional value (e.g., quality, variety) of food for mass feeding sites. Plans included utilizing inventory from USDA Foods for food assistance programs when responding to a disaster.  Needs/preferences were briefly mentioned when sourcing food for individuals with disabilities, mothers, and infants. Religious, cultural, ethical, local, organic preferences were not directly addressed. |
| **Texas** | The disaster plan integrated donation assistance by outlining coordination roles among local food banks, food pantries, school meals, and other in-kind food programs within the Feeding America network and the Texas Food Bank Network (TFBN). These roles involved emergency mass feeding preparation, distribution, and soliciting donated groceries.  Regarding supply and provision, the plans included donation assistance from neighboring states. The Texas Multi-Agency Feeding Task Force (TMAFTF) collaborated on planning and deployment during disasters. The plan also incorporated public and private partnerships among NGOs, food banks, churches, academic institutions, and service providers to supplement the USDA food inventory. Supply and provision were outlined in a three-phase timeline, detailing the immediate, sustained, and long-term provision of food. | The disaster plan integrated financial food aid (e.g. DSNAP, WIC, FNS waivers) to address affordability concerns, income changes, and child nutrition programs resulting from disasters. Communication and access to disaster-related assistance were available through local county officials and the 2-1-1 Texas Information and Referral Network.  The plan addressed the physical accessibility of transportation, distribution networks, and the number of food sources. Fixed Feeding Operations at shelters and designated points and Mobile Feeding Operations were deployed to areas of need, coordinated by the TMAFTF. TFBN coordinated with the TMAFTF to distribute food supplies from local pantries. TMAFT coordinated feeding to alleviate duplication of resources and worked with vendors for efficient delivery of food services. NGOs and Texas Voluntary Organizations Active in Disaster (TXVOAD) provided bulk distribution services and transportation.  Social accessibility regarding trust, worry, fear, stigma, or coping behaviors were not directly addressed. | Infrastructure such as an individual’s ability to access kitchen facilities, equipment, space, and ingredients were not directly addressed.  Self-efficacy in food preparation and cooking ability was briefly mentioned, with the restoration of utilities allowing residents to cook meals in their homes and purchase food in stores. Knowledge of food usage, desire, and confidence in meal preparation were not directly addressed. | | The disaster plan did not detail nutritional value but mentioned TMAFTF’s collaboration on feeding menus. It briefly mentioned providing food and beverages suitable for individuals with dietary restrictions.  Needs/preferences such as medical, religious, cultural, ethical, local, organic, or food that can be stored and prepared were not directly addressed. |
| **Louisiana** | The disaster plan integrated donation assistance by outlining coordination roles among local food banks, food pantries, school meals, and other in-kind food programs within the Feeding Louisiana regional food banks and their network of partner agencies. These roles involved emergency mass feeding preparation, distribution, and soliciting donated groceries.  Regarding supply and provision, the plan emphasized comprehensive inventory management, including daily maintenance of stock status reports. Specific food service providers and the active involvement of restaurants were highlighted for their capacity to supply a significant number of in-kind meals per day. The supply and provision strategy were outlined in a three-phase timeline, detailing the immediate, sustained, and recovery stages of food provision. The plan also included donation assistance from neighboring states, with the Louisiana Mass Feeding Taskforce (LMFTF) collaborating on planning and deployment during disasters. | The disaster plan integrated financial food aid (e.g. DSNAP, TEFAP, WIC, FNS waivers) to address affordability concerns, income changes, and child nutrition programs resulting from disasters. Communication and access to disaster-related assistance were available through the Louisiana Department of Agriculture and Forestry (LDAF).  The plan recognized capacity challenges in the physical accessibility of distribution networks and supply. It emphasized the need to coordinate efforts and resources from Federal, State, and NGOs to address mass feeding needs. The plan addressed the number of food sources throughout, considering aspects of transportation and obstacles to mass feeding capabilities, such as the inability to access normal food distribution sites (e.g., grocery stores). Its focus was on enhancing food resource distribution in response to challenges like transportation issues, the impact of multiple disasters, and potential loss of feeding infrastructure.  Social accessibility regarding trust, worry, fear, stigma, or coping behaviors was not directly addressed. | Instead of focusing on restoring individuals' infrastructure or self-efficacy in food preparation, disaster plans integrated mobile kitchens and equipment. Infrastructure such as kitchen facilities and equipment were addressed with the use of mobile facilities like kitchens and delivery vehicles in cases where food infrastructure was compromised. Space and ingredients were not directly addressed.  Self-efficacy in food preparation and cooking ability, their knowledge of food usage, or their desire and confidence in meal preparation were not directly addressed. | The disaster plan referenced the Louisiana Office of Public Health (OPH) Sanitarian Services, which played a role in maintaining inspections from food providers as an "approved source." According to the Louisiana Administrative Code, an "approved supplier" was defined as producers, manufacturers, distributors, or food establishments acceptable based on conformity with applicable laws, public health principles, and industry standards. Plans mentioned adhering to nutritional guidelines for feeding operations, ensuring alignment with established standards, and the capability to meet specific dietary requirements to the best extent possible.  Needs/preferences such as religious, cultural, ethical, local, organic, or food that can be stored and prepared were not directly addressed. | |
| **Puerto Rico** | Donation and assistance efforts observed in media depicted severe food shortages and reliance on relief supplies. Initiatives like emergency kitchens, food trucks, canning sessions, and cash donations were noted as effective means of addressing immediate food needs and supporting local recovery efforts.  Media coverage highlighted FEMA's mishandling of commodity distribution, including lost shipments and delayed deliveries, with significant backlogs at the main distribution port in Jacksonville, Florida. The Jones Act exacerbated these challenges by requiring U.S.-built ships for shipments to Puerto Rico, while Puerto Rico's inventory tax further restricted companies from stockpiling inventory. In response, partnerships were forged between local food banks, relief agencies, and restaurants to address supply and provision needs following the hurricane. | Media reports highlighted challenges in accessing financial food aid, as the Nutrition Assistance Program (NAP) operated under capped funding and provided a fixed amount of money regardless of need, unlike DSNAP. Additionally, power and communication outages hindered residents' ability to make electronic purchases.  Media reports highlighted physical access issues, noting that much of the food remained stuck at the port and was unable to be distributed to more accessible food distribution sites.  Media coverage delved into the broader narrative of social accessibility factors, encompassing people's worries and fears about accessing food and the prolonged effects on the food supply resulting from the hurricane. | Media reports highlighted how power outages hampered individuals' ability to access kitchen facilities, equipment, space, and ingredients.  Self-efficacy in food preparation and cooking ability, their knowledge of food usage, or their desire and confidence in meal preparation were not directly addressed. | Reports indicated vagueness regarding the type and quality of FEMA's food assistance, raising concerns about the nutritional value, including the inclusion of junk food items.  Needs/preferences such as medical, religious, cultural, ethical, local, organic, or food that can be stored and prepared were not directly addressed. | |
| **U.S. Virgin Islands** | Media coverage of donation and assistance efforts emphasized addressing immediate food needs, with local organizations such as churches and relief groups playing a pivotal role in ensuring food availability.  However, media reports also highlighted FEMA's mishandling of commodity distribution, including lost shipments and delayed deliveries. In response, FEMA established distribution sites across the U.S. Virgin Islands. On St. Thomas, the primary industrial port faced space limitations for unloading shipping containers. Addressing the needs of populations across three islands presented unique challenges, with obstacles persisting due to limited port capacity and resource competition between relief and commercial supply chains. | Media accounts highlighted how the U.S. Virgin Islands benefited from access to DSNAP nutritional programs, but power and communication outages hindered residents' ability to make electronic purchases.  Media reports highlighted physical access issues, noting that much of the food remained stuck at the port and was unable to be distributed to more accessible food distribution sites.  Media coverage delved into the broader narrative of social accessibility factors, encompassing people's worries and fears about accessing food and the prolonged effects on the food supply resulting from the hurricane. | Media reports highlighted how power outages hampered individuals' ability to access kitchen facilities, equipment, space, and ingredients.  Self-efficacy in food preparation and cooking ability, their knowledge of food usage, or their desire and confidence in meal preparation were not directly addressed. | Nutritional value (e.g., quality, variety) of food was not directly addressed.  Needs/preferences such as medical, religious, cultural, ethical, local, organic, or food that can be stored and prepared were not directly addressed. | |
